# Analysis of Sub-Lethal Toxicity of Perfluorooctane Sulfonate (PFOS) to *Daphnia magna* Using ^1^H Nuclear Magnetic Resonance-Based Metabolomics

**DOI:** 10.3390/metabo7020015

**Published:** 2017-04-14

**Authors:** Martha N. Kariuki, Edward G. Nagato, Brian P. Lankadurai, André J. Simpson, Myrna J. Simpson

**Affiliations:** Environmental NMR Centre and Department of Physical and Environmental Sciences, University of Toronto Scarborough, 1265 Military Trail, Toronto, ON M1C1A4, Canada; martha.kariuki@gmail.com (M.N.K); edward.nagato@gmail.com (E.G.N.); brianlanka@gmail.com (B.P.L); andre.simpson@utoronto.ca (A.J.S.)

**Keywords:** environmental metabolomics, environmental stressors, bioindicators, PFOS mode of action, sub-lethal toxicity, aquatic ecosystem health

## Abstract

^1^H nuclear magnetic resonance (NMR)-based metabolomics was used to characterize the response of *Daphnia magna* after sub-lethal exposure to perfluorooctane sulfonate (PFOS), a commonly found environmental pollutant in freshwater ecosystems. Principal component analysis (PCA) scores plots showed significant separation in the exposed samples relative to the controls. Partial least squares (PLS) regression analysis revealed a strong linear correlation between the overall metabolic response and PFOS exposure concentration. More detailed analysis showed that the toxic mode of action is metabolite-specific with some metabolites exhibiting a non-monotonic response with higher PFOS exposure concentrations. Our study indicates that PFOS exposure disrupts various energy metabolism pathways and also enhances protein degradation. Overall, we identified several metabolites that are sensitive to PFOS exposure and may be used as bioindicators of *D. magna* health. In addition, this study also highlights the important utility of environmental metabolomic methods when attempting to elucidate acute and sub-lethal pollutant stressors on keystone organisms such as *D. magna*.

## 1. Introduction

Perfluorinated chemicals have many industrial applications due to their unique surfactant properties as well as their remarkable stability that results from high energy carbon-fluorine bonds. Perfluorinated chemicals are used in insecticide formulas, lubricants, polymer additives, protective coatings for clothing and other apparels [1,2,3,4]. Their increased use and exceptional stability has led to their global distribution and persistence in the environment [4]. Perfluorooctane sulfonate (PFOS) is a commonly found perfluorinated compound that has been observed in lakes, oceans, soils, plants, and in living beings including humans [2,4,5]. PFOS concentrations in the environment are typically observed in the ng/L–µg/L range but can be found in the mg/L range, especially near point sources of PFOS pollution [6,7]. PFOS is ubiquitously found in the environment, and it continues to bioaccumulate in a range of organisms [6]. Furthermore, information regarding the toxicity of PFOS at sub-lethal concentrations is lacking. PFOS studies in mammalian laboratory experiments have revealed substantial weight loss, alteration in lipid metabolism, liver toxicity, pancreatic damage, and mortality with exposure to high concentrations [1,2,3,8,9,10]. Because of the presence of PFOS and its related family of compounds [6], it is important to understand its toxicity to various organisms, particularly at low concentrations and at the metabolite level, in an attempt to mitigate the unfavorable outcomes from exposure.

Studies have indicated the persistence of PFOS and other perfluorinated compounds in lakes, rivers, and oceans along with its bioaccumulation in aquatic organisms such as fish [3,11,12,13]. *Daphnia magna* is a small crustacean, which is a prevalent keystone species in various freshwater aquatic ecosystems and has been used to assess pollutant toxicity on aquatic life [14,15,16,17]. They occupy an intermediate position in the food web, are ubiquitous, highly sensitive to toxicants, easy to culture, fast growing, and have a short lifespan, making them suitable organisms for ecotoxicity tests and the analysis of aquatic ecological food webs [18,19]. Because of this, environmental metabolomic studies using *D. magna* and related species have increased substantially [18,20,21,22,23,24,25,26,27]. However, very little is known about the mechanisms of sub-lethal PFOS toxicity and how sub-lethal exposure alters the *D. magna* metabolome. 

With PFOS exposure, *D. magna* experience reduced reproduction and an accelerated heartrate [28,29]. In addition, PFOS, when present in a pollutant mixture, can alter the toxicity of other pollutants and exacerbate the overall toxic response [30]. Furthermore, the impairments caused by PFOS can last for several generations [29], suggesting that chronic exposure will severely alter *Daphnia* population dynamics and possibly higher trophic levels [19]. Wagner et al. [27] compared metabolic profiles of juvenile and adult daphnids after an acute exposure (48 h) to sub-lethal PFOS. Although the juvenile and adult daphnids responded differently, metabolic profiles shifted significantly with exposure [27]. As such, further information is required to determine the toxic mode of action (MOA) of PFOS in daphnids over a range of sub-lethal concentrations. This study focuses on the metabolic responses of adult *D. magna* with PFOS exposure using ^1^H nuclear magnetic resonance (NMR) analysis of polar metabolites [31]. NMR-based metabolomics is widely used to study the toxicity of a range of environmental pollutants to various organisms that exist in different ecosystems [32,33,34,35,36,37,38,39]. NMR is commonly used because it is non-targeted, highly reproducible, and provides a quantitative overview of metabolites present in a tissue extract or biofluid [33,40,41]. Previous studies have used NMR-based metabolomics to characterize the sub-lethal toxicity of *Daphnia* species [18,22,23,27,42] to various anthropogenic stressors. Detailed NMR mapping of the *D. magna* metabolome has been performed recently and metabolite assignments have been verified using two-dimensional NMR experiments [31]. In this study, we build upon previous method development and toxicity studies to investigate how PFOS alters the metabolome of *D. magna*. The lethal concentration that results in 50% mortality of the population (LC_50_) for PFOS is reported as 130 mg/L [43] for *D. magna*. We therefore focused on acute exposures (48 h) using 15, 30, 45, and 60 mg/L of PFOS to test how the metabolome is altered over a range of sub-lethal concentrations. 

## 2. Results

### 2.1. Principal Component Analysis (PCA) and Partial Least Squares (PLS) Regression

**Figure 1:**
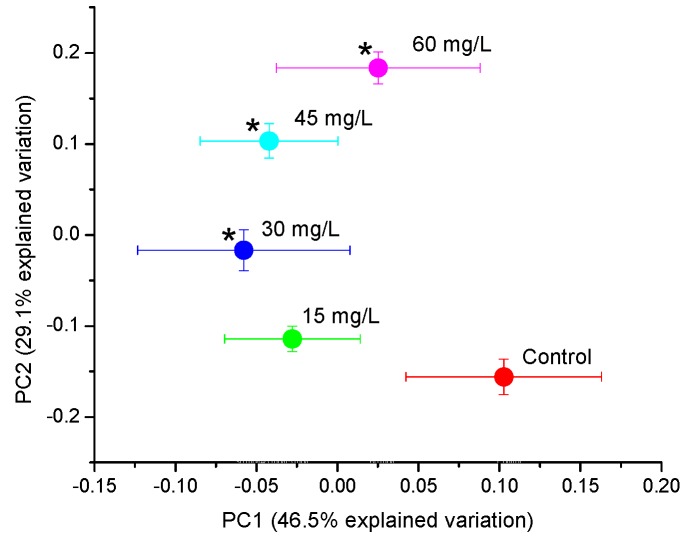
Averaged scores plot from principal component analysis (PCA) of PFOS-exposed and non-exposed (control) metabolic profiles. Statistically significant separation from the control (*p* < 0.05) is indicated by an asterisk (*).

To compare the metabolic profiles of the PFOS exposed daphnia to the unexposed group, individual and average PCA scores plots were constructed (Figure 1, Supplementary Materials Figures S1–S4). Both the individual and the averaged plots revealed a progressive separation of metabolites with increasing PFOS concentration particularly along PC2 (Figure 1, Supplementary Materials Figures S1–S4). Also illustrated by PC2 was that the lowest exposure concentration (15 mg/L) did not have a statistically significant separation from the control; however the 30 mg/L, 45 mg/L, and 60 mg/L exposures showed statistically significant separation. The 15 mg/L exposure concentration did illustrate some variation from the control (Supplementary Materials Figure S1) however its response was less prominent than those of the other concentrations suggesting that this exposure concentration did not elicit a distinct metabolite change from the control. This concentration-dependent change in metabolite response between the control and exposed groups, showed that the 60 mg/L exposure concentration induced the largest metabolite response in *D. magna* (Figure 1). Thus it is evident that PFOS imparts an increasing concentration-dependent metabolite response in *D. magna* over the range of exposure concentrations used.

**Figure 2:**
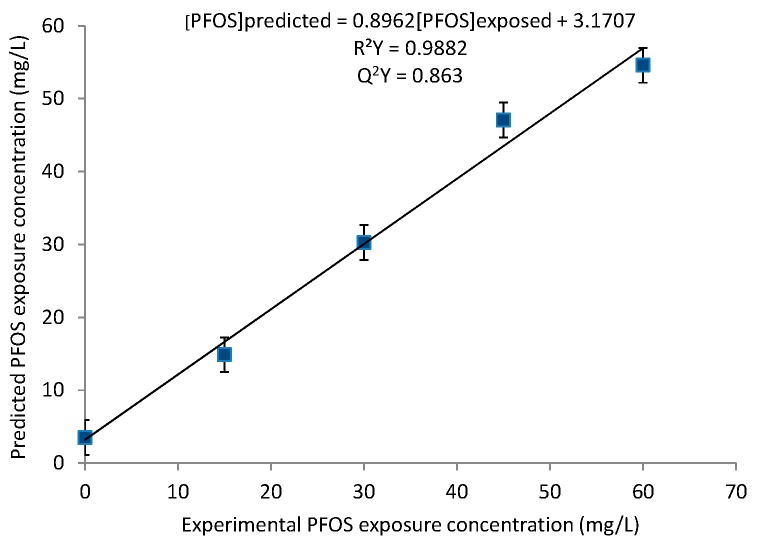
Partial Least Squares (PLS) regression model derived from the leave-one-out cross-validation procedure, illustrating the average predictions of PFOS exposure concentrations using the bucketed ^1^H NMR spectra. The relationship between the predicted and experimental data is linearly correlated with a R^2^Y of 0.9882 and a Q^2^Y of 0.863.

To further test the significance and strength of the relationship between the PFOS exposure concentrations and metabolic response of the *D. magna*, PLS regression modeling was performed. The PLS regression model revealed a strong linear correlation between the PFOS exposure concentrations and *D. magna* metabolic response with a R^2^Y value of 0.9882 and a Q^2^Y value of 0.863 (Figure 2), indicating that the model is robust [44]. The positive linear regression between the experimental and predicted values highlights the strong correlation between the metabolic response and the PFOS exposure concentration. Furthermore, the PLS regression results are consistent with the increasing separation with higher PFOS exposure concentrations observed with PCA (Figure 1). These results illustrate the suitability of *D. magna* as a model organism for toxicity testing of PFOS and possibly other perfluorinated chemicals, as well as the application of these experimental results in predictive statistical models that may aid in obtaining toxicity thresholds in aquatic environments [45].

### 2.2. Metabolite Changes with PFOS Exposure

**Figure 3:**
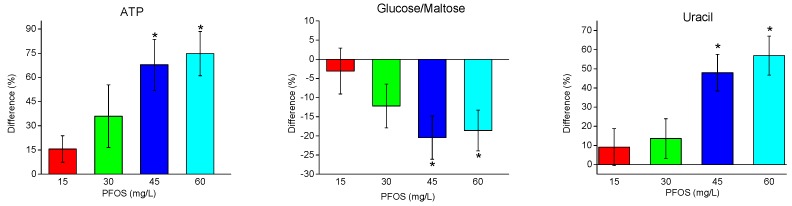
Percent changes in metabolites related to energy and enzyme regulation (relative to the control) with increasing PFOS exposure. Asterisks indicate significant differences (*p* < 0.05).

**Figure 4:**
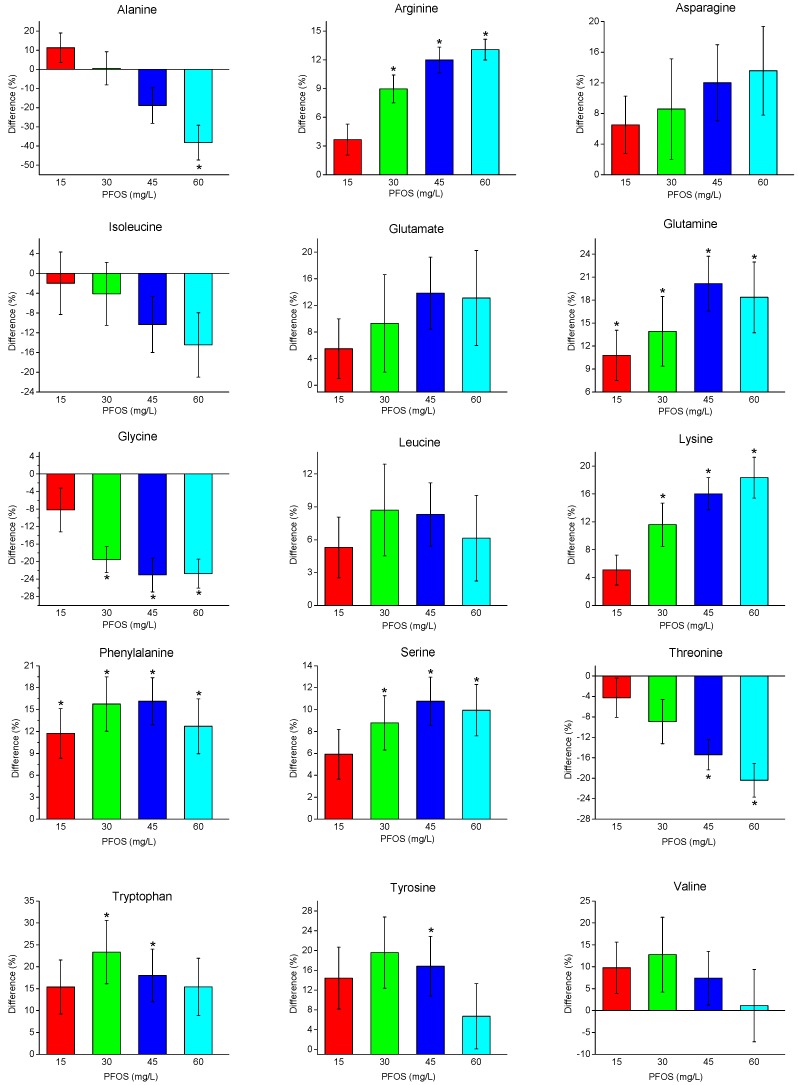
Percent changes in amino acid concentrations (relative to the control) with increasing PFOS exposure concentration (mg/L). Asterisks indicate significant differences (*p* < 0.05).

PCA loadings plots (Supplementary Materials Figure S5) were used to identify which NMR spectral buckets were responsible for the observed separation within the PCA scores plots (Figure 1). Metabolite identification was performed using published ^1^H NMR metabolite resonances as well as previous work in our laboratory with NMR-based metabolomics of *D. magna* [31,46,47]. Metabolites that were observed to make a significant contribution to the separation observed in the PCA scores plot included a number of amino acids, the sugar glucose, the nucleobase uracil, and the energy molecule adenosine triphosphate (ATP). Overall, 18 metabolites were identified and quantified from the ^1^H NMR spectra. The percent changes in these metabolites, relative to the control, with increasing PFOS concentration are shown in Figure 3 and Figure 4. Interestingly, as with the PCA and PLS data, most metabolites are observed to be changing in relation to the PFOS exposure concentration. Both ATP and uracil increased significantly with higher PFOS exposure (Figure 3). Alternatively, glucose/maltose reserves decreased with increasing PFOS exposure (Figure 3).

Of the amino acids measured, three different trends are observed (Figure 4): (i) increasing amino acids with increasing PFOS exposure, (ii) decreasing amino acids with increasing PFOS exposure, and (iii) non-monotonic variation in amino acid levels with increasing PFOS exposure. Several amino acids (arginine, asparagine, and lysine) increased with increasing PFOS exposure. This observation is consistent with the PLS results which suggested a linear concentration-dependent metabolic response with PFOS exposure. In contrast, alanine, isoleucine, and threonine decreased with increasing PFOS exposure. Several other amino acids, which either increased or decreased, appeared to plateau with higher PFOS concentrations. These included: glutamate, glutamine, glycine, leucine, phenylalanine, serine, tryptophan, tyrosine, and valine. Collectively, these results highlight the sensitivity of ^1^H-NMR based metabolomics which demonstrates that metabolites can behave uniquely, as a function of the toxic MOA, in response to sub-lethal pollutant exposure.

## 3. Discussion

Overall, our results show that acute (48 h) sub-lethal PFOS exposure elicited both linear and non-monotonic shifts in *D. magna* polar metabolites. The averaged PCA scores plots show increasing separation with increasing exposure to PFOS (Figure 1) and the linearity of the overall metabolic response is further supported by the PLS results (Figure 2). However, closer inspection of the metabolite changes (Figure 3 and Figure 4) shows varying responses of individual metabolites. In regard to molecules related to energy, ATP was observed to increase with increasing PFOS exposure. Alternatively, glucose was observed to decrease with increasing PFOS concentrations. Glucose is a primary substrate for glycolysis that leads to the release of the cells energy currency, ATP [48,49]. The decrease in glucose and subsequent increase in ATP suggests that PFOS likely enhances energy metabolism in *D. magna*, driving the breakdown of glucose via glycolysis which leads to the observed increase in ATP. Another metabolic process that produces ATP is the beta-oxidation of fatty acids, which generates a high yield of ATP through oxidative phosphorylation [50,51,52]. Interestingly, other studies on the common carp, tilapia, and earthworms illustrated an inhibition of ATP synthesis as a result of PFOS exposure [47,53,54]. This suggests that PFOS may have multiple MOAs depending on the range of exposure concentrations and the test organism. Qi et al. [45] reported that PFOS adsorbs onto an organism’s membrane through both ionic and hydrophobic interactions and this may alter the behavior of PFOS in an organism, leading to a variation in sensitivity and observed MOA upon exposure. 

The nucleobase uracil was also observed to increase with increasing PFOS exposure concentrations. Uracil plays various roles in metabolic activity, one of which is the regulation of coenzymes including the role of converting glucose to galactose [55,56]. The observed increase in uracil, with increasing PFOS exposure, suggests an attempt at enzyme regulation in response to the deregulation being caused by PFOS toxicity. Both Jeong et al. [29] and Liang et al. [28] reported that sub-lethal PFOS exposure reduces enzyme activity in *D. magna*. As mentioned previously, a decrease in glucose was observed with increasing PFOS concentration along with an increase in ATP. Glycogen, a polymeric form of glucose, is generally considered the most prominent fuel source in daphnia [57]. A decrease in glucose could lead to a relatively equivalent increase in ATP as glucose plays a primary role in ATP synthesis; however the positive percent change in ATP was observed to be ~80% and the negative percent change in glucose, was ~20% (Figure 3). This suggests that there may be other metabolic pathways being used for the synthesis of ATP in *D. magna.* Because of the observed perturbation of metabolites involved with energy metabolism, the MOA of PFOS likely targets metabolic processes involved with energy metabolism. However because of the broadness of this area of energy synthesis, further work needs to be performed to better deduce the exact pathway(s) for *D. magna*.

The amino acids alanine, isoleucine, glycine, and threonine were observed to decrease linearly with increasing PFOS concentrations. This suggests either the depletion of these amino acids or a decrease in their production in response to PFOS toxicity. Alanine initially increased upon exposure to PFOS at 15 mg/L, though it decreased with higher PFOS concentrations. Alanine plays a key role in the glucose-alanine cycle which allows for its conversion to glucose as an extra energy source [58,59,60]. Furthermore, glucose and the glucogenic amino acids such as isoleucine, glycine, and threonine were observed to decrease with increasing PFOS concentrations. This observed decrease in glycogen stores upon PFOS exposure has also been observed in the common carp (*Cyprinus carpio*) [53], tilapia (*Oreochromis niloticus*) [54], and earthworms (*Eisenia fetida*) [47], and likely inhibited ATP synthesis. The regulation of energy use and expenditure is of key importance in aquatic invertebrates when adapting to an external stressor [61]. This suggests that PFOS causes an increase in energy metabolism by interfering with key metabolic pathways hence fueling the need for more ATP that is required for detoxification and cell regulation. However, this occurs at a cost by negatively affecting other energy processes required for survival, namely gluconeogenesis [53]. PFOS exposure has also been attributed to the production of fatty acid oxidation enzymes. The structure of PFOS resembles that of naturally present fatty acids, allowing it to bind to proteins such as serum albumin, leading to an increase in beta-oxidation of fatty acids through peroxisome proliferation and this has been observed in various fish species, rats, mice, chicken liver, and salmon [62,63,64,65,66,67]. The production of these fatty acid oxidation enzymes requires the use of free amino acids, which can also explain the observed decrease in some amino acids. 

Arginine, asparagine, and lysine increased proportionally with PFOS exposure. Glutamate, glutamine, leucine, phenylalanine, serine, tryptophan, tyrosine, and valine also increased but these increases were non-monotonic with increasing PFOS exposure (Figure 4). The non-monotonic behavior was more prevalent with the 45 and 60 mg/L PFOS exposure concentrations which may represent a threshold in the MOA where these metabolites plateaued. The observed shifts in amino acids can be attributed to protein degradation, which increases the concentration of free amino acids, and/or shifts to key metabolic processes such as fatty acid synthesis and energy synthesis. For example, one of the biological roles of phenylalanine is its conversion to another essential amino acid tyrosine which is then used to produce dopamine and other signaling molecules involved with the regulation of growth, metabolism, stress response, and pigmentation [68,69]. The disruption in normal cell function caused by PFOS toxicity consequently induces an increase in detoxification and maintenance via the regulation of growth and other metabolic process [53], which is consistent with the observed changes in phenylalanine and subsequent tyrosine production. The non-monotonic behavior observed in the amino acid phenylalanine, may be attributed to saturation of the phenylalanine-4-hydroxylase enzyme necessary for the metabolic conversion of phenylalanine to tyrosine, which allows for the continued metabolic activity seen in tyrosine as it aids in the production of signaling molecules such as melanin and dopamine [70,71]. The amino acid leucine is used in the regulation of cell growth, formation of sterols, and in the synthesis of muscle protein [72,73]. In this study, leucine and phenylalanine have a similar response behavior with increasing PFOS exposure and may be attributed to increased pollutant stress in the daphnia leading to the saturation of enzymes such as acetolactate synthase necessary for the production of leucine [74]. Arginine, which was the third amino acid to lack concentration-dependence upon increasing PFOS exposure, also plays a significant role in immune function, energy metabolism and in the release of other key hormones required for regulation [75,76]. The observed initial increase in arginine release suggests an increase in immune function as a consequence of the increasing presence of the PFOS toxicant, which begins to level off as a result of the metabolic burden caused by the increasing PFOS concentration. This observed behavior in the three key amino acids—phenylalanine, leucine, and arginine—suggests that PFOS has a MOA that targets key metabolic pathways involved in growth, hormone regulation, energy metabolism, and stress response, which in turn halts the immune function responses of *D. magna*.

## 4. Materials and Methods 

### 4.1. Culturing and Maintenance of Daphnia magna

*Daphnia magna* were purchased from Ward Science Canada and cultured according to Environment Canada guidelines in the laboratory environment [77]. The cultures were maintained at a room temperature of 20 ± 1 °C with a photoperiod of 16h light/8h dark in glass vessels containing de-chlorinated and aerated tap-water (hardness ~120 mg/L). Daphnia were fed with a 50:50 algal mixture of *Raphidocelis subcapitata* and *Chlorella vulgaris*. Media was changed three times a week with an algae mixture supplementation on alternate days (every 24 h). For sub-lethal exposure experiments, only fully-grown adults (>14 days old) were used to minimize variations in metabolite profiles due to variation in life stage [27]. 

### 4.2. Sub-Lethal Exposure Experiments

*Daphnia magna* were exposed to sub-lethal concentrations of PFOS (heptadecafluoroocate sulfonic acid potassium salt, 98%, Sigma-Aldrich, Oakville, ON, Canada) for 48 h. The sub-lethal concentration was selected based on a reported measure of acute toxicity that results in 50% mortality in the population (LC_50_ value) of 130 mg/L [43]. A preliminary PFOS exposure experiment was performed to ensure survival, leading to increasing experimental exposure concentrations of 15, 30, 45, and 60 mg/L of PFOS. Each exposure concentration comprised of 10 populations (n = 10) made of 12 individual adult daphnids. In addition, an unexposed control population (n = 10, 12 individuals each) was included as a reference comparison to the PFOS exposed populations. Based on previous experiments [31], each sample was comprised of 12 adult daphnids. This allows for the ~1 mg dry mass (7–8 daphnids) that is required for extraction to obtain a suitable ^1^H NMR spectrum. 

PFOS was dissolved in Milli-Q water (Millipore Corporation, Etobicoke, ON, Canada) at a stock concentration of 300 mg/L and serial dilutions were performed to achieve the appropriate exposure concentration. Each 2 L exposure vessel was initially filled with 1 L of medium (see Section 2.2) after which the daphnia were added to allow for acclimatization. The PFOS toxicant was then added appropriately for each exposure concentration after which the vessels were topped with de-chlorinated/aerated tap-water (see Section 4.1). Concentrations of stock solutions and exposure medium solutions at 0, 24, and 48 h were confirmed using an Agilent Technologies 6500 series Accurate-Mass Quadrupole Time-of-Flight (QTOF) liquid chromatography/mass spectrometry (LC/MS) operating with a Z-spray ESI source working in negative mode. LC/MS analysis did not reveal any PFOS degradation or losses during the course of the experiment (data not shown). The 48 h exposures were maintained at the same room temperature and photoperiod as the stock *D. magna* culture (see Section 4.1).

### 4.3. Metabolite Extraction and Analysis by ^1^H NMR Spectroscopy

Previous research in our laboratory developed and tested different extraction methods for *D. magna* and also carried out detailed identification of polar metabolites using both one-dimensional and two-dimensional NMR spectroscopy [31]. A range of polar metabolites were extracted using a D_2_O-based buffer from *D. magna* based on the optimized method reported in Nagato et al. [31]. After exposure, the daphnids were removed from the medium and flash-frozen in liquid nitrogen and lyophilized to limit enzyme activity. Samples were then stored frozen (−20 °C) until extraction. To each 1 ± 0.1 mg dry mass of daphnia, 40 µL of a D_2_O 0.2 M phosphate buffer (NaH_2_PO_4_.H_2_O, 99.3%, Fisher Canada) adjusted to *p*D = 7.4 with NaOD (30% w/w in 99.5% D_2_O, Cambridge Isotope Laboratories Inc.) was added. The phosphate buffer also included a preservative of 0.1% w/v sodium azide (95% purity, Sigma Aldrich) and an internal calibrant of 10 mg/L sodium 2,2-dimethyl-2-silapentane-5-sulfonate (DSS, 97% Sigma Aldrich, [31]). The samples were each vortexed for 45 s, immediately followed by sonication for 15 min and then centrifugation for 20 min at 14,000 rpm (~15,000 g). The supernatant was then pipetted directly from the centrifuge tubes into 1.7 mm capillary NMR tubes (Wilmad-LabGlass, Vineland, NJ, USA) for ^1^H NMR analysis.

*Daphnia magna* extracts were analyzed using a Bruker BioSpin Avance III 500MHz spectrometer and a ^1^H-^13^C-^15^N 1.7 mm microprobe fitted with an actively shielded Z gradient (Bruker BioSpin, Rheinstetten, Germany). The ^1^H NMR experiments were performed using Presaturation Using Relaxation Gradients and Echoes (PURGE) water suppression which was developed specifically for environmental metabolomic applications [78]. Studies have demonstrated that water suppression using PURGE is superior to other available methods for the acquisition of ^1^H NMR spectra [79,80]. NMR spectra were collected using 256 scans with a relaxation time of 3 s and 64 k time domain points [31]. The spectra were apodized by multiplication with an exponential decay corresponding to 0.3 Hz line broadening and a zero filling factor of 2 [31]. All the spectra were manually phased and calibrated to the methyl protons of the trimethylsilyl group of the DSS (δ = 0.00 ppm).

### 4.4. Data Analysis

^1^H NMR spectra were bucketed using the AMIX statistical tool (v. 3.9.7, Bruker BioSpin, Rheinstetten, Germany). The spectral region between 0.5 and 10 ppm was divided into buckets of 0.02 ppm width for a total of 475 buckets. The spectral region that corresponds to the residual H_2_O/HOD signals (between 4.7 and 4.9 ppm) was excluded during bucketing, and the spectra were normalized to the sum of total intensities. The bucketed data was then used to create principal component analysis (PCA) scores plots. Average PCA scores plots and their associated standard errors were also constructed for each of the four exposure concentrations to examine possible trends related to any concentration-dependent relationships. An analysis of variance (ANOVA) with a Bonferroni posthoc test was performed using an in-house R script to assess statistical significance in separation between groups in the PCA scores plots. Metabolites contributing to separation between the PCA scores for the control and exposed daphnia were identified using PCA loadings plots, which are plots that are a representation of each of the 475 buckets and their relative contribution in the separation between groups. 

Metabolite assignment in the ^1^H NMR spectra was based on previous work that has identified metabolites in *D. magna* using both one-dimensional and two-dimensional NMR techniques [31], available NMR spectra through the Madison Metabolomics Consortium Database [46], and published resonances [47]. Two example ^1^H NMR spectra are shown in Supplementary Materials Figure S6. The percentage change in metabolite signal intensity upon exposure was then obtained by subtracting the control bucket values from each of the exposure concentrations and dividing the value by the control. ANOVA with a Bonferroni posthoc test was used to determine statistically significant changes with PFOS exposure.

## 5. Conclusions

This study illustrated the ability of ^1^H-NMR based metabolomics to elucidate significant metabolic changes in the *D. magna* metabolome upon exposure to sub-lethal PFOS concentrations. We identified several concentration-dependent relationships with some metabolites, with increasing PFOS concentrations. The sub-lethal toxic MOA in *D. magna* upon PFOS exposure includes a disruption to energy dynamics as well as protein degradation. This is consistent with other studies that focused on other environmentally-relevant organisms such as various fish species and earthworms [47,53,62]. Our study also revealed that PFOS may have multiple metabolite-specific MOAs that are concentration-dependent. Although this study only focused on four PFOS concentrations, we were able to observe trends in certain key metabolites involved in similar metabolic processes allowing us to enhance our understanding of the MOA of PFOS. Thus, further work utilizing a wider range of PFOS concentrations, specifically with *D. magna*, are needed to better characterize the precise MOA and explore why some metabolites exhibit non-monotonic behavior. Our study focused particularly on the polar metabolites that change in *D. magna* upon exposure to PFOS; however the mode of action illustrated by PFOS is likely to influence non-polar metabolites particularly because of its reported disruption on fatty acid metabolism [64,65,66,67]. Thus, future work should also include the exploration of non-polar metabolites perturbed in *D. magna* upon exposure to PFOS.

## Supplementary Materials

The following are available online at www.mdpi.com/2218-1989/7/2/15/s1, Figure S1: Principal component analysis (PCA) scores plot of *D. magna* exposed to 15 mg/L PFOS compared to controls (unexposed daphnids). Each data point represents an individual metabolic profile measured by ^1^H NMR spectroscopy; Figure S2: Principal component analysis (PCA) scores plot of *D. magna* exposed to 30 mg/L PFOS compared to controls (unexposed daphnids). Each data point represents an individual metabolic profile measured by ^1^H NMR spectroscopy; Figure S3: Principal component analysis (PCA) scores plot of *D. magna* exposed to 45 mg/L PFOS compared to controls (unexposed daphnids). Each data point represents an individual metabolic profile measured by ^1^H NMR spectroscopy; Figure S4: Principal component analysis (PCA) scores plot of *D. magna* exposed to 60 mg/L PFOS compared to controls (unexposed daphnids). Each data point represents an individual metabolic profile measured by ^1^H NMR spectroscopy; Figure S5: Principal component analysis (PCA) loadings plot of *D. magna* exposed to PFOS compared to controls (unexposed daphnids). Some metabolites are labelled based on published studies and metabolite database information: 1 = leucine, 2 = isoleucine, 3 = threonine, 4 = alanine, 5 = arginine, 6 = lysine, 7 = glutamate, 8 = glycine, 9 = tryptophan, 10 = glucose/maltose, 11 = uracil, 12 = phenylalanine, and 13 = ATP; Figure S6: Two example ^1^H NMR spectra acquired for *Daphnia magna* polar metabolites. The overlay shows differences in the metabolite resonance intensities which are summarized in the principal component analysis (PCA) scores plots. Full metabolite assignment and more details about the NMR extraction method are available in Nagato et al. [31].
