# Supplementary material for: Analysis of Sub-Lethal Toxicity of Perfluorooctane Sulfonate (PFOS) to Daphnia magna Using 1H Nuclear Magnetic Resonance-Based Metabolomics
Source: Metabolites. 2017 Apr 14;7(2):15. doi: 10.3390/metabo7020015 (PMC5487986; doi:10.3390/metabo7020015)
Supplement: Supplementary file 1 [file metabolites-07-00015-s001.pdf]

Supplementary Materials: Analysis of sub-lethal toxicity of perfluorooctane sulfonate (PFOS) to *Daphnia magna* using ^{1}H nuclear magnetic resonance-based metabolomics

Martha N. Kariuki, Edward G. Nagato, Brian P. Lankadurai, André J. Simpson, and Myrna J. Simpson

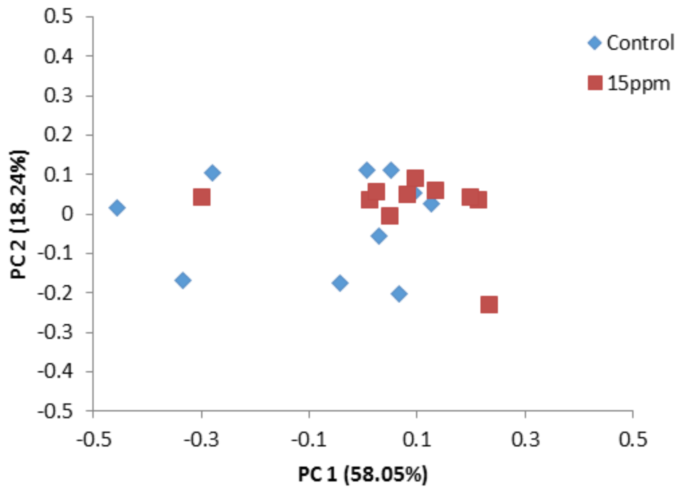

Figure S1. Principal component analysis (PCA) scores plot of *Daphnia magna* exposed to 15 mg/L PFOS compared to controls (unexposed daphnids). Each data point represents an individual metabolic profile measured by ^{1}H NMR spectroscopy.

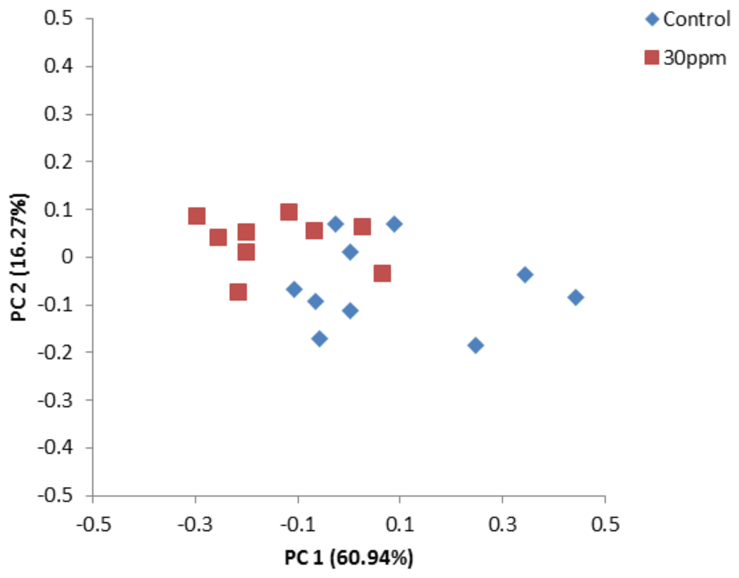

Figure S2. Principal component analysis (PCA) scores plot of *Daphnia magna* exposed to 30 mg/L PFOS compared to controls (unexposed daphnids). Each data point represents an individual metabolic profile measured by ^{1}H NMR spectroscopy.

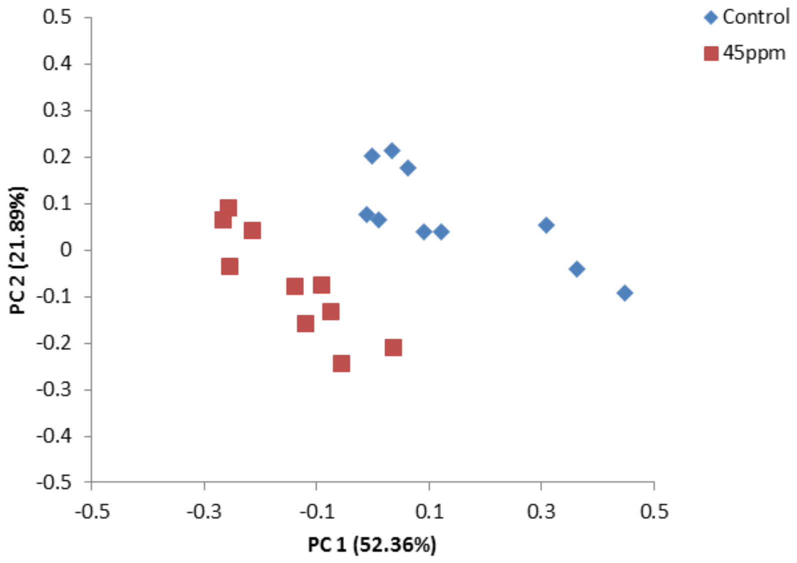

Figure S3. Principal component analysis (PCA) scores plot of *Daphnia magna* exposed to 45 mg/L PFOS compared to controls (unexposed daphnids). Each data point represents an individual metabolic profile measured by ^{1}H NMR spectroscopy.

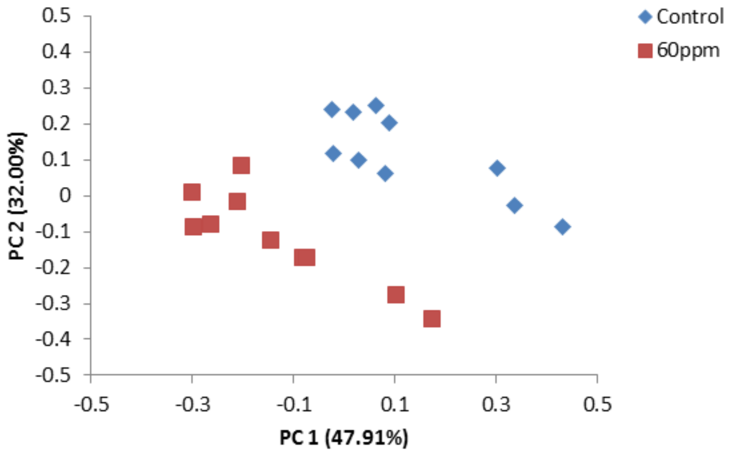

Figure S4. Principal component analysis (PCA) scores plot of *Daphnia magna* exposed to 60 mg/L PFOS compared to controls (unexposed daphnids). Each data point represents an individual metabolic profile measured by ^{1}H NMR spectroscopy.

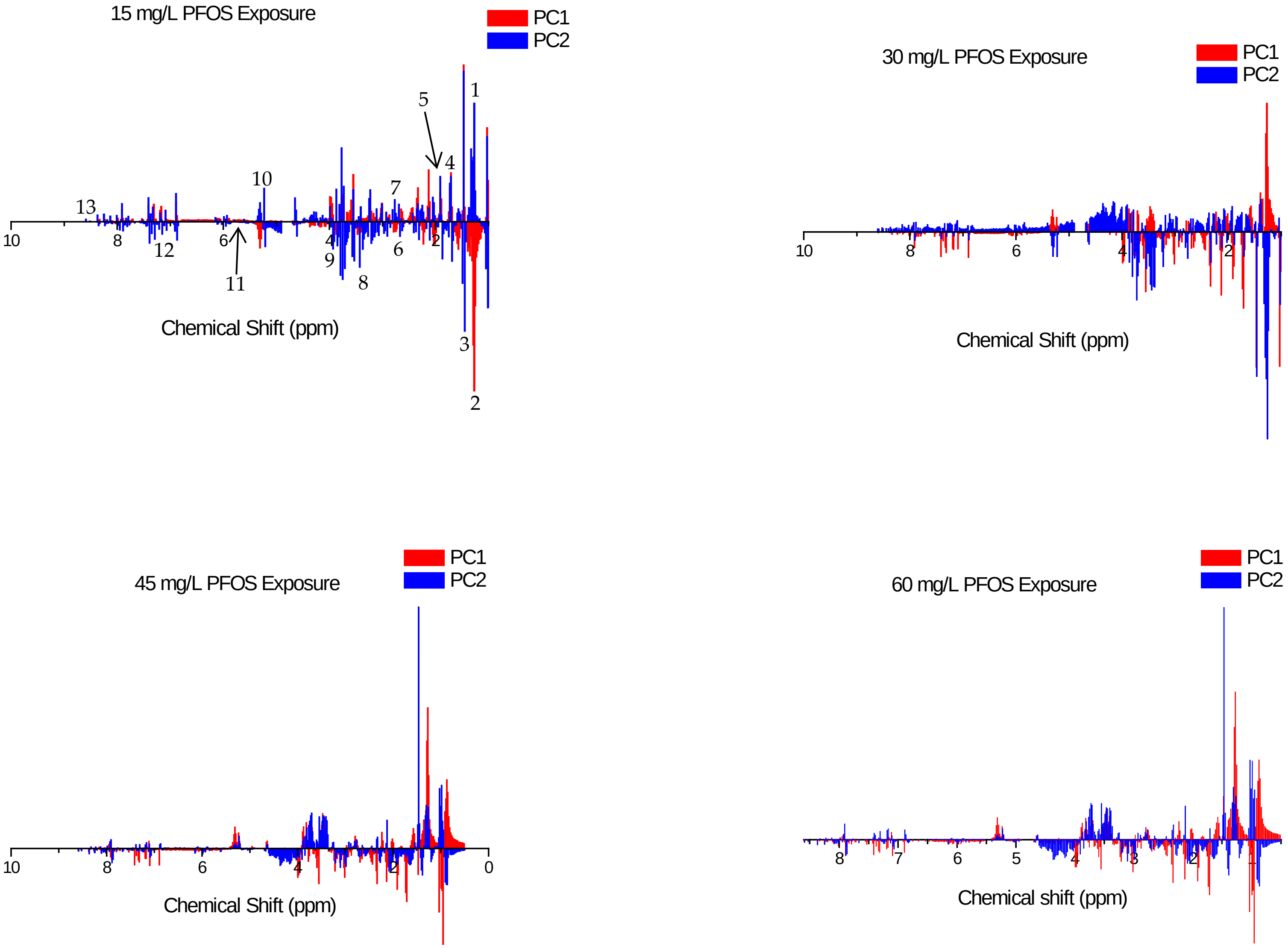

Figure S5. Principal component analysis (PCA) loadings plot of *Daphnia magna* exposed to PFOS compared to controls (unexposed daphnids). Some metabolites are labelled based on published studies and metabolite database information [1-3]: 1 = leucine, 2= isoleucine, 3=threonine, 4= alanine, 5= arginine, 6= lysine, 7= glutamate, 8= glycine, 9= tryptophan, 10= glucose/maltose, 11= uracil, 12= phenylalanine, and 13= ATP.

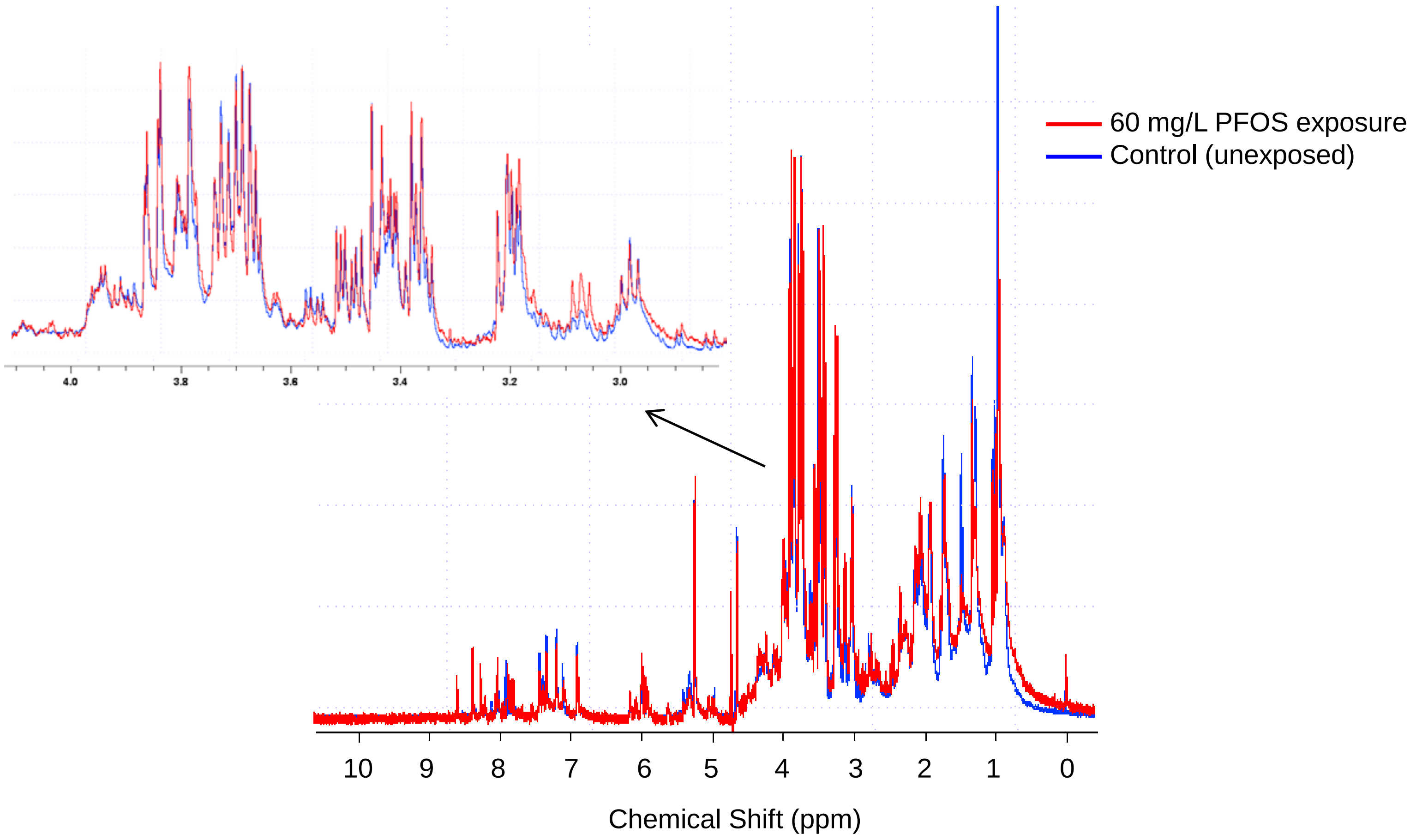

Figure S6: Two example ¹H NMR spectra acquired for *Daphnia magna* polar metabolites. The overlay shows differences in the metabolite resonance intensities which are summarized in the principal component analysis (PCA) scores plots. Full metabolite assignment and more details about the NMR extraction method are available in Nagato et al. [3].

References

1. Cui, Q.; Lewis, I.A.; Hegeman, A.D.; Anderson, M.E.; Li, J.; Schulte, C.F.; Westler, W.M.; Eghbalnia, H.R.; Sussman, M.R.; Markley, J.L. Metabolite identification via the Madison Metabolomics Consortium Database. *Nat Biotechnol* **2008**, *26*, 162-164.
2. Lankadurai, B.P.; Furdui, V.I.; Reiner, E.J.; Simpson, A.J.; Simpson, M.J. ^{1}H NMR-based metabolomic analysis of sub-lethal perfluorooctane sulfonate exposure to the earthworm, *Eisenia fetida*, in soil. *Metabolites* **2013**, *3*, 718-740.
3. Nagato, E.G.; Lankadurai, B.P.; Soong, R.; Simpson, A.J.; Simpson, M.J. Development of an NMR microprobe procedure for high-throughput environmental metabolomics of *Daphnia magna*. *Magn. Reson. Chem.* **2015**, *53*, 743-753.
